# Supplementary material for: SARS‐CoV‐2 sensing by RIG‐I and MDA5 links epithelial infection to macrophage inflammation
Source: EMBO J. 2021 Jul 2;40(15):e107826. doi: 10.15252/embj.2021107826 (PMC8209947; doi:10.15252/embj.2021107826)
Supplement: Supplementary file 2 — Appendix [file EMBJ-40-e107826-s002.pdf]

Appendix Table of contents

Appendix Figure S1

Appendix Figure S2

Appendix Table S1-S7

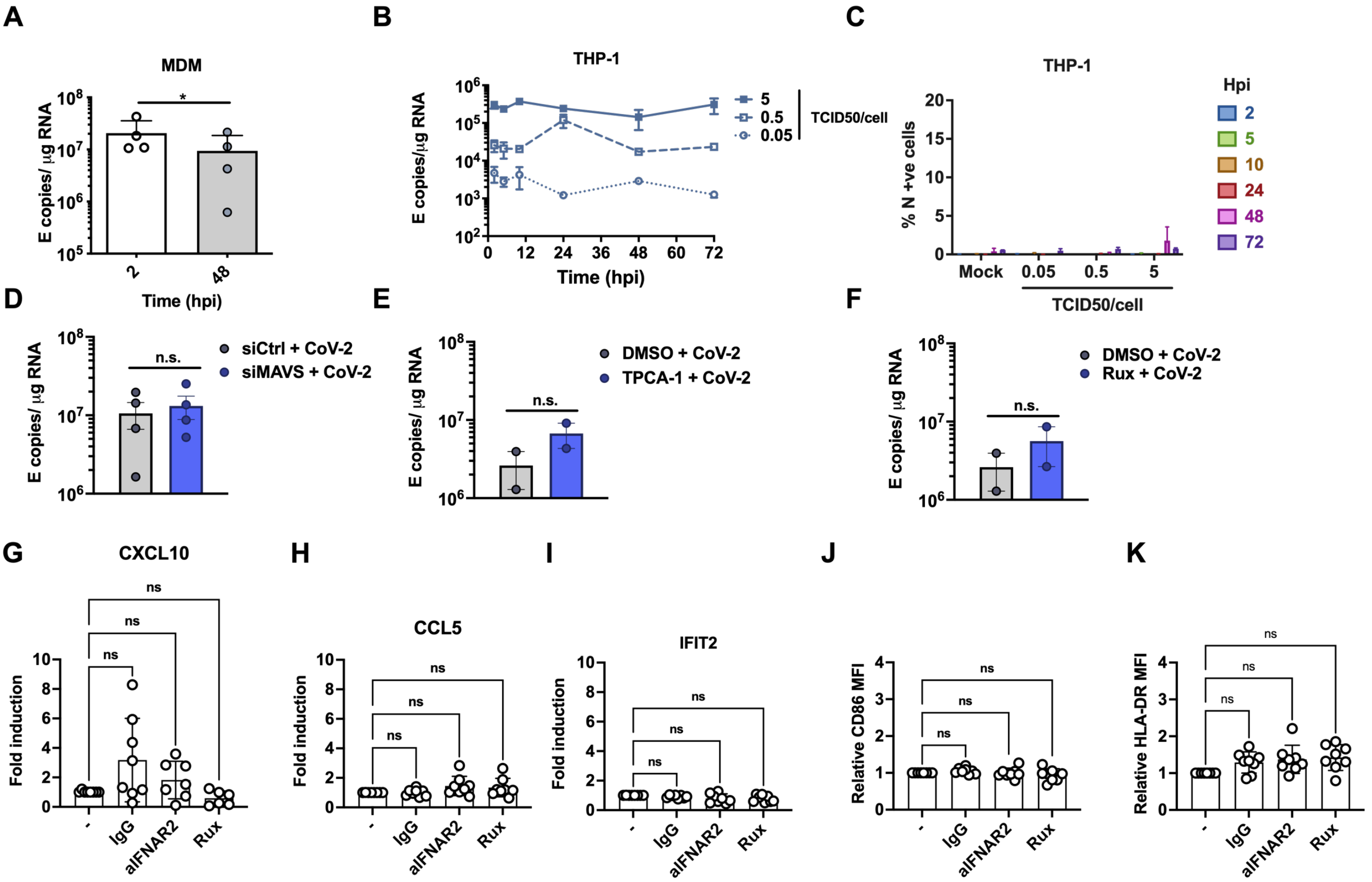

Appendix Figure S1. (**A**) MDM were exposed to SARS-CoV-2 for 2 or 48h and viral genomic and subgenomic E RNA measured (RT-qPCR), means shown +/- SEM, n=4. Statistical comparison between 2 and 48h by paired two-tailed t test, *, p<0.05. (**B, C**) PMA-differentiated THP-1 macrophages were exposed to SARS-CoV-2 for 72h measuring (**B**) viral genomic and subgenomic E RNA (qRT-PCR) or (**C**) N protein +ve cells (flowcytometry). Mean+/-SEM, n=2. (**D-F**) Detection of viral genomic and subgenomic E RNA extracted from MDM exposed for 48 h to SARS-CoV-2-containing conditioned medium from Calu-3 cells infected in the presence or absence of (D) MAVS depletion by siRNA, (E) 10 µM TPCA-1 or (F) 2 µM Ruxolitinib. Mean +/- SEM n=2-4. Statistical comparison by paired two-tailed t test, n.s. : non significant. **(G-K)** MDM were treated with either anti-IFNAR antibody (2.5ug/ml), an isotype control IgG antibody (IgG, 2.5ug/ml), ruxolitinib (2 µM), or mock treated during 6 h of exposure to CoM from uninfected, unmodified Calu-3 cells, before washing and measuring MDM gene expression (G, H, I), and MDM activation markers (J,K) by flowcytometry 48 h later. Both gene expression and relative MFI are compared to mock-treated MDM exposed to CoM from uninfected Calu-3s cells. Statistical testing by one-way paired ANOVA, comparing treated MDMs to untreated control by Dunnett's multiple comparison test, ns : non significant.

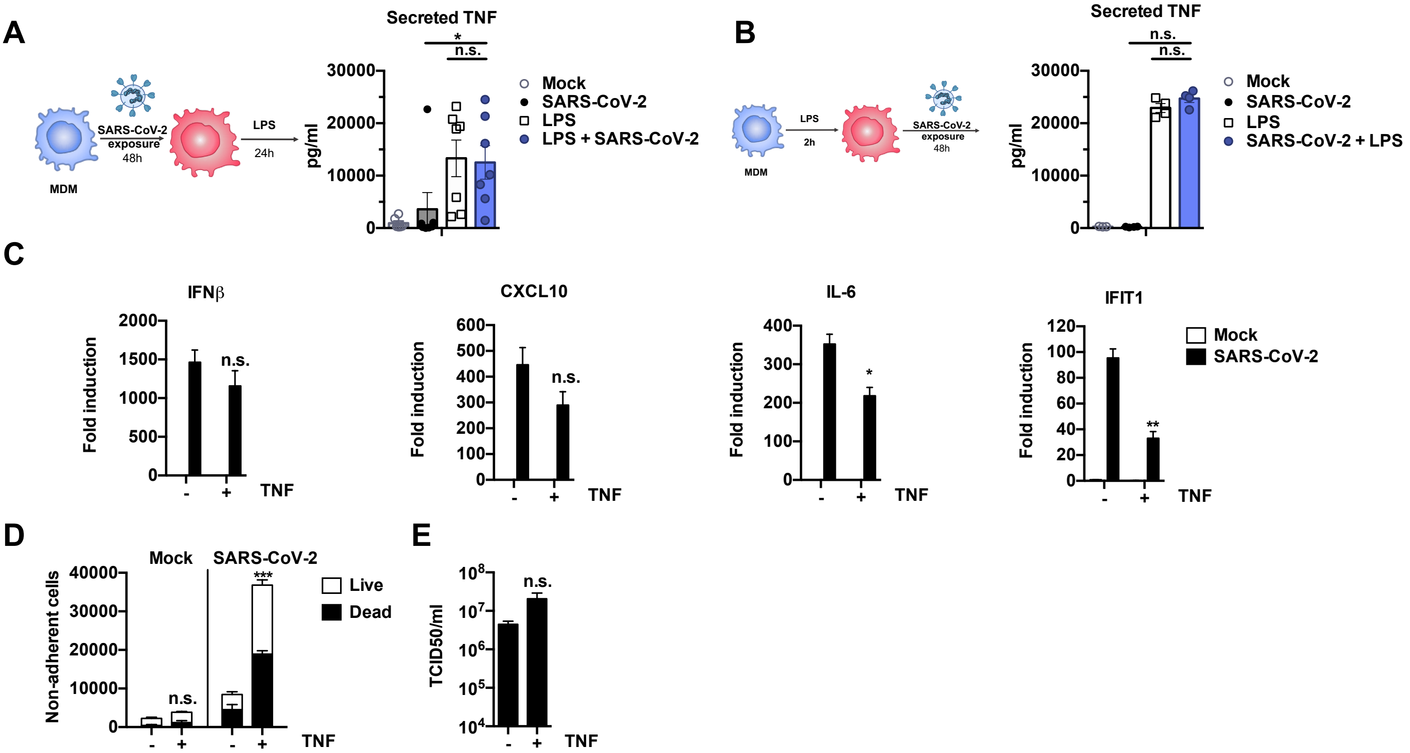

Appendix Figure S2. (**A-B**) Schematic of experimental design showing timing and order of exposure of MDM to LPS and SARS-CoV-2 (as in figure 6), and measurement of TNF (ELISA). Groups were compared as indicated by Wilcoxon matched-pairs signed rank test, *, $p<0.05$. Mean+/-SEM, n=4-7. (**C-E**) SARS-CoV-2 infection of Calu-3 cells (MOI 0.04 $TCID50_{VERO}$/cell) in the presence or absence of 10 ng/ml TNF added at the time of infection. SARS-CoV-2 infected TNF + and – groups were compared by two tailed t test. *, $p<0.05$; **, $p<0.01$; n.s., non-significant. Mean +/- SEM shown, n=3. **(C)** Fold gene induction of *IFN*β, *CXCL10, IL-6,* and *IFIT1* at 24hpi. (**D**) Quantification of Live/Dead staining of non-adherent cells recovered from culture supernatants at 24hpi. (**E**) Virus titres in Calu-3 supernatants at 24 hpi ($TCID50_{VERO}$/cell).

**Appendix Table S1. Analysis parameters for detection of SARS-CoV-2 infection by intracellular nucleocapsid protein in Calu-3 cells.**	
**Viral infection (Intracellular Granules Module, 10X/0.4 NA)**	**Analysis Parameters:**
Nucleus : Nucleus Smooth	1.9
Nucleus : Nucleus Background Subtraction	175
Nucleus : Nucleus Intensity Threshold	310
Nucleus : Nucleus Max Patch Size	100
Nucleus : Nucleus Maximum Area	800 mic.
Nucleus : Nucleus Minimum Area	32 mic.
Cell : Cell Smooth	5
Cell : Cell Background Subtraction	2000
Cell : Cell Intensity Threshold	5000
Granules : Granules Smooth	5
Granules : Granules Background Subtraction	45
Granules : Granules Intensity Threshold	930
Granules : Granules Maximum Area	1000 mic.
Granules : Granules Max Patch Size	20
Granules : Granules Minimum Area	10 mic.
Population: Infected: Mean Granule Inensity	≥1000
Output	Cell Count, Nuclear Area, Nuclear Intensity (DAPI), Cell Area (CellMask), Granule Area, Granule Intensity (N-Protein)

Appendix Table S2. Analysis parameters for IRF3/NFkB nuclear intensity during SARS-CoV-2 infection in Calu-3 cells.	
**IRF3/NFkB Nuclear Intensity (Intranuclear Foci Module, 40X/0.75NA)**	**Analysis Parameters:**
Nucleus : Nucleus Smooth	1
Nucleus : Nucleus Background Subtraction	109.8
Nucleus : Nucleus Intensity Threshold	150
Nucleus : Nucleus Maximum Merge Area	60
Nucleus : Nucleus Minimum Area	30
Nucleus : Nucleus Maximum Area	900
Foci : Foci Smooth	1
Foci : Foci Background Subtraction	105
Foci : Foci Intensity Threshold	2170
Foci : Foci Maximum Merge Area	105 mic.
Foci : Foci Minimum Area	30 mic.
Foci : Foci Maximum Area	2000 mic.
Output:	Cell Count, Nuclear Area, Nuclear Intensity (DAPI), Nuclear Intensity of Marker Protein (IRF3/NFkB), Foci Area, Foci Intensity (N-Protein)

Appendix Table S3. Analysis parameters for RNA FISH detection during SARS-CoV-2 infection in Calu-3 cells.	
**RNA FISH analysis (Mitochondria Module, 40X/0.75 NA)**	**Analysis Parameters:**
Nucleus : Smooth	1.2
Nucleus : Background Subtraction	68
Nucleus : Intensity Threshold	220
Nucleus : Maximal area for merging	30
Nucleus : Minimum Area	57 mic.
Nucleus : Maximum Area	800 mic.
Cell : Smooth	2
Cell : Intensity Threshold	2500
Mitochondria : Background Subtraction	108.8
Mitochondria : Minimum Length	1
Mitochondria : Typical Width	1
Mitochondria : Intensity Threshold	2000
Mitochondria : Maximal area for merging	1
Mitochondria : Minimum Area	1 mic.
Mitochondria : Maximum Area	121 mic.
Output:	Cell Count, Nuclear Area, Nuclear Intensity (DAPI), Cell Area (CellMask), Cellular Intensity of Marker Protein(IL6/IFIT1/GAPDH), Mitochondria Area/ Intensity (N-Protein)

Appendix Table S4. Total population cell numbers imaged for each condition.

	Total population (hours post infection)					
**SARS-CoV-2 infection (10X)**	**2hpi**	**6hpi**	**10hpi**	**24hpi**	**48hpi**	**72hpi**
Mock	19884	23937	22564	29058	36589	40971
MOI0.04	16877	21856	21103	23128	10954	6811
MOI0.4	18171	21811	21671	24401	14784	8751
MOI2	16507	22215	21397	22324	12199	6584
**Nuclear accumulation: NFkB (40X)**						
Mock	4208	4732	2561	5811	6547	7404
MOI0.0004	1308	1322	1477	1250	949	1120
MOI0.004	1490	1481	1583	1461	1073	643
MOI0.04	3580	4445	4044	4594	2565	1706
MOI0.4	4136	4521	4245	4425	2533	2102
MOI2	2186	3402	2785	3360	2393	1309
**Nuclear accumulation: IRF3 (40X)**						
Mock	3718	4347	3607	4602	6205	8140
MOI0.0004	1512	1110	1363	1156	1285	1029
MOI0.004	1290	1250	1310	1489	797	445
MOI0.04	4125	4389	4515	4910	2465	2140
MOI0.4	4602	3966	4782	4148	2572	1728
MOI2	2417	3287	3213	2834	2035	1620
**RNA-FISH IL-6 (40X)**						
MOI 0.4	-	3677	4331	4203	-	-
**RNA-FISH GAPDH (40X)**						
MOI 0.4	-	3876	3620	4276	-	-

Appendix Table S5. Numbers of N positive cells imaged for NFkB and IRF3 nuclear translocation in each condition.

Nuclear accumulation: NFkB (40X)				N+ cells		
	2hpi	6hpi	10hpi	24hpi	48hpi	72hpi
MOI0.0004	0	0	0	0	356	276
MOI0.004	0	0	4	56	116	19
MOI0.04	0	18	71	1875	1309	643
MOI0.4	0	37	358	1874	1423	791
MOI2	0	146	446	2048	1373	509
**Nuclear accumulation: IRF3 (40X)**						
MOI0.0004	0	0	0	0	301	67
MOI0.004	0	0	0	86	159	27
MOI0.04	0	18	71	1875	1309	643
MOI0.4	0	34	360	2096	1323	607
MOI2	0	123	557	1669	1162	592

Nuclear accumulation: NFkB (40X)				Total cells		
MOI0.0004	1308	1322	1477	1250	949	1120
MOI0.004	1490	1481	1583	1461	1073	643
MOI0.04	3580	4445	4044	4594	2565	1706
MOI0.4	4136	4521	4245	4425	2533	2102
MOI2	2186	3402	2785	3360	2393	1309
**Nuclear accumulation: IRF3 (40X)**						
MOI0.0004	1512	1110	1363	1156	1285	1029
MOI0.004	1290	1250	1310	1489	797	445
MOI0.04	4125	4389	4515	4910	2465	2140
MOI0.4	4602	3966	4782	4148	2572	1728
MOI2	2417	3287	3213	2834	2035	1620

Appendix Table S6. Numbers of N negative cells imaged for NFkB and IRF3 nuclear translocation in each condition.

Nuclear accumulation: NFkB (40X)				N-ve cells		
	2hpi	6hpi	10hpi	24hpi	48hpi	72hpi
MOI0.0004	1308	1322	1477	1250	593	844
MOI0.004	1490	1481	1579	1405	957	624
MOI0.04	3580	4427	3973	2719	1256	1063
MOI0.4	4136	4484	3887	2551	1110	1311
MOI2	2186	3256	2339	1312	1020	800
**Nuclear accumulation: IRF3 (40X)**						
MOI0.0004	1512	1110	1363	1156	984	962
MOI0.004	1290	1250	1310	1403	638	418
MOI0.04	4125	4371	4444	3035	1156	1497
MOI0.4	4602	3932	4422	2052	1249	1121
MOI2	2417	3164	2656	1165	873	1028

Appendix Table S7. Numbers of N negative cells imaged for NFkB and IRF3 nuclear translocation in each condition.

Nuclear accumulation: NFkB (40X)	2hpi	6hpi	10hpi	24hpi	48hpi	72hpi
Mock rep1	2981	3532	1499	4410	4866	5843
Mock rep2	1227	1200	1062	1401	1681	1561
Total	4208	4732	2561	5811	6547	7404

Nuclear accumulation: IRF3 (40X)	2hpi	6hpi	10hpi	24hpi	48hpi	72hpi
Mock rep1	2675	3139	2522	3288	4647	6011
Mock rep2	1043	1208	1085	1314	1558	2129
Total	3718	4347	3607	4602	6205	8140
