# Supplementary material for: SARS‐CoV‐2 sensing by RIG‐I and MDA5 links epithelial infection to macrophage inflammation
Source: EMBO J. 2021 Jul 2;40(15):e107826. doi: 10.15252/embj.2021107826 (PMC8209947; doi:10.15252/embj.2021107826)
Supplement: Supplementary file 3 — Expanded View Figures PDF [file EMBJ-40-e107826-s003.pdf]

Expanded View Figures

Figure EV1. SARS-CoV-2 replication in Caco-2 cells does not induce an innate response.

A Immunoblot detecting ACE2 expression in epithelial (Detroit 562, Beas2B, Calu-3, Caco-2), endothelial (HULEC5a) and PMA-differentiated THP-1 cells. b-Actin is detected as loading control.

B *ACE2*, *TMPRSS2* and *TMPRSS4* gene expression in cell lines and primary monocyte-derived macrophages (MDM). Relative expression normalised to *GAPDH* mean ± SEM $n = 2$.

C–G Measurements of replication and innate immune induction in Caco-2 intestinal epithelial cells infected with SARS-CoV-2 at MOI 0.08, 0.4 or 2 $TCID50_{VERO}$/cell. Mean ± SEM, $n = 2$. (C) SARS-CoV-2 genomic and subgenomic E RNAs (qRT-PCR). (D) Infectious virus released from cells in (C) determined by TCID50 on Vero.E6 cells, mean ± SEM $n = 2$. (E) Quantification of N staining from cells in (C) by flow cytometry. Mean percentage of N-positive of all live-gated cells ± SEM, $n = 2$. (F) Representative example of immunofluorescence staining of N protein (green) after SARS-CoV-2 infection of Caco-2 at MOI 0.4 $TCID50_{VERO}$/cell, at time points shown. Nuclei (DAPI, blue), cell mask (red). (G) Fold induction of interferon and interferon-stimulated genes (ISG) of infections in (C) at 24 h and 72 hpi at MOIs $TCID50_{VERO}$/cell 0.08, 0.4 or 2, $n = 2$.

H Fold induction of ISG and cytokine gene expression in Caco-2 cells in response to innate immune activation with poly(I:C), R837 and LPS for 24 h, $n = 2$.

I Fold induction of ISG and cytokine gene expression in Calu-3 cells in response to innate immune activation with poly(I:C) (+/− transfection, TF), R837 and LPS for 24 h, $n = 2$. Mean ± SEM.

 © 2021 The Authors

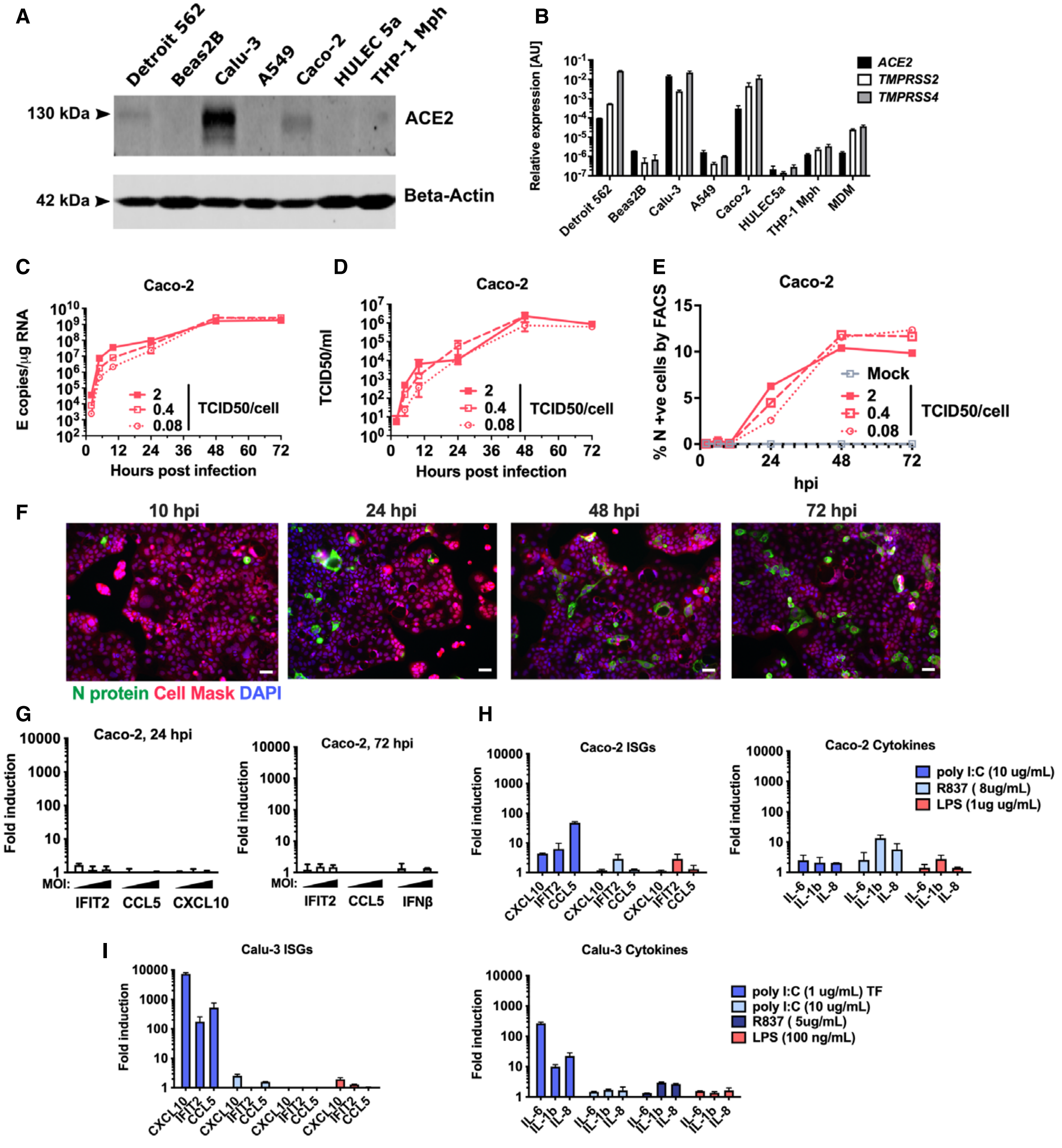
A
Detroit 562
Beas2B
Calu-3
A549
Caco-2
HULEC 5a
THP-1 Mph
130 kDa
ACE2
42 kDa
Beta-Actin
B
Relative expression [AU]
ACE2
TMPRSS2
TMPRSS4
Detroit 562
Beas2B
Calu-3
A549
Caco-2
HULEC5a
THP-1 Mph
MDM
C
Caco-2
E copies/µg RNA
2
0.4
0.08
TCID50/cell
Hours post infection
D
Caco-2
TCID50/ml
2
0.4
0.08
TCID50/cell
Hours post infection
E
Caco-2
% N +ve cells by FACS
Mock
2
0.4
0.08
TCID50/cell
hpi
F
10 hpi
24 hpi
48 hpi
72 hpi
N protein Cell Mask DAPI
G
Caco-2, 24 hpi
Fold induction
MOI:
IFIT2
CCL5
CXCL10
Caco-2, 72 hpi
Fold induction
MOI:
IFIT2
CCL5
IFNβ
H
Caco-2 ISGs
Fold induction
CXCL10
IFIT2
CCL5
Caco-2 Cytokines
Fold induction
poly I:C (10 ug/mL)
R837 (8ug/mL)
LPS (1ug ug/mL)
IL-6
IL-1b
IL-8
I
Calu-3 ISGs
Fold induction
CXCL10
IFIT2
CCL5
Calu-3 Cytokines
Fold induction
poly I:C (1 ug/mL) TF
poly I:C (10 ug/mL)
R837 (5ug/mL)
LPS (100 ng/mL)
IL-6
IL-1b
IL-8

Figure EV1.

Figure EV2. SARS-CoV-2 replicates rapidly in Calu-3 cells and induces a delayed innate response.

A–C Measurements of viral replication in Calu-3 lung epithelial cells infected with SARS-CoV-2 at MOIs 0.0004, 0.004, 0.04 or 0.4 $TCID50_{VERO}$/cell, $n = 3$. (A) Replication of SARS-CoV-2 genomic and subgenomic E RNAs (qRT-PCR). (B) Quantification of N protein-positive cells from (A) by flow cytometry. Mean percentage of N +ve of all live-gated cells. (C) Infectious virus released from cells in (A) determined by TCID50 on Vero.E6 cells.

D Fold induction of chemokines from infections in (Fig 1) (*CCL5*, *CXCL10*, *CCL2*, *CCL3*), cytokines (*IL-8*, *IL-6*, *IL-1*β, *IL1*α, *TNF*), Interferons (*IFN*β, *IFN*λ*1*, *IFN*λ*3*) and ISGs (*IFIT2*, *MX1*, *ISG56*) at 24 hpi in Calu-3 cells infected at MOIs 0.08, 0.4 or 2 $TCID50_{VERO}$/cell, $n = 2$.

E Fold induction of *IFIT2*, *CCL5*, *CXCL10*, *IL-6*, *IFN*β, *IFN*λ*1*, *IFN*λ*3* in Calu-3 cells at MOI 0.08 or 2 $TCID50_{VERO}$/cell each overlaid with SARS-CoV-2 E (qRT-PCR), $n = 2$.

F Fold induction of *CXCL10*, *IL-6* and *IFIT1* in SARS-CoV-2-infected Calu-3 cells from (A) at MOIs 0.0004, 0.004, 0.04 or 0.4 $TCID50_{VERO}$/cell, $n = 3$.

G SARS-CoV-2 infection (MOIs 0.04 $TCID50_{VERO}$/cell) in Calu-3 cells after addition of 10 ng/ml IFNβ, IFNλ1, IFNλ2 or IFNγ before or after infection at time points shown, measured by E RNA copies, N-positive cells (relative to untreated infection) and released virus as $TCID50_{VERO}$/ml, all measured at 24 hpi. Treatments were compared to untreated SARS-CoV-2 infected Calu-3 cells by *t*-test. $*P < 0.05$; $**P < 0.01$; $***P < 0.001$ or exact *P*-value are shown. Mean ± SEM shown, $n = 3$.

H Fold induction of *CXCL10* and *IFIT1* in interferon-treated Calu-3 cells at 24 h. Means ± SEM, $n = 3$.

 © 2021 The Authors

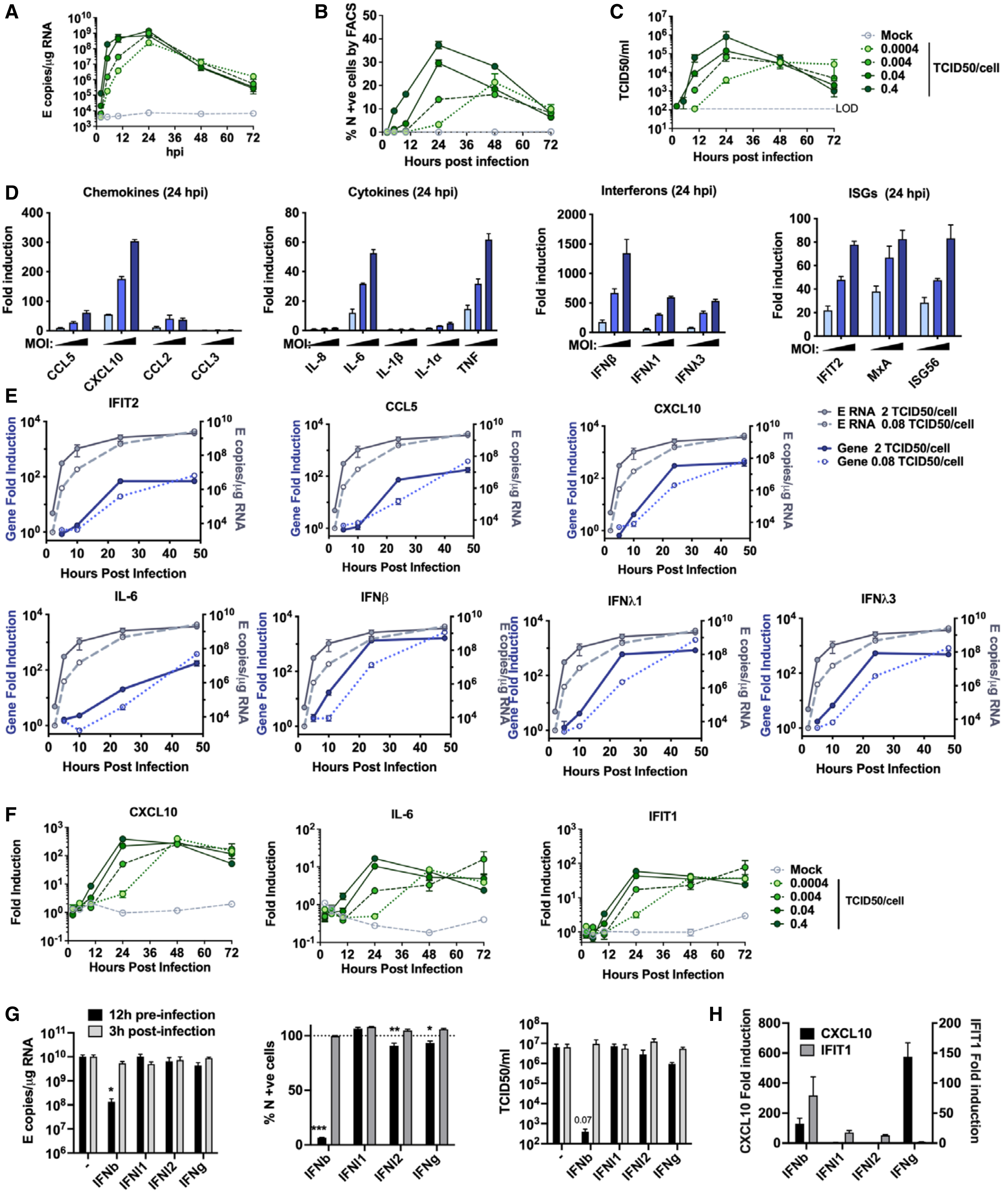

A
E copies/μg RNA
hpi
B
% N +ve cells by FACS
Hours post infection
C
TCID50/ml
LOD
Hours post infection
Mock
0.0004
0.004
0.04
0.4
TCID50/cell
D
Chemokines (24 hpi)
Cytokines (24 hpi)
Interferons (24 hpi)
ISGs (24 hpi)
Fold induction
MOI:
CCL5
CXCL10
CCL2
CCL3
IL-8
IL-6
IL-1β
IL-1α
TNF
IFNβ
IFNλ1
IFNλ3
IFIT2
MxA
ISG56
E
IFIT2
CCL5
CXCL10
IL-6
IFNβ
IFNλ1
IFNλ3
Gene Fold Induction
E copies/μg RNA
Hours Post Infection
E RNA 2 TCID50/cell
E RNA 0.08 TCID50/cell
Gene 2 TCID50/cell
Gene 0.08 TCID50/cell
F
CXCL10
IL-6
IFIT1
Fold Induction
Hours Post Infection
Mock
0.0004
0.004
0.04
0.4
TCID50/cell
G
12h pre-infection
3h post-infection
E copies/μg RNA
% N +ve cells
TCID50/ml
IFNb
IFNl1
IFNl2
IFNg
0.07
H
CXCL10
IFIT1
CXCL10 Fold induction
IFIT1 Fold induction

Figure EV2.

Figure EV3. NF-κB and IRF3 translocation in SARS-CoV-2-infected cells.

Single-cell analysis time course quantifying the integrated nuclear intensity of NF-κB p65 or IRF3 in SARS-CoV-2-infected Calu-3 cells at MOI 2, 0.4 or 0.04 $TCID50_{VERO}$/cell or mock infected as labelled. At all time points, nuclear intensities of NF-κB or IRF3 in nucleocapsid protein-positive infected cells (blue) and N-ve cells (grey) are shown. Nuclear intensities of uninfected cells (Mock) at 24 h are shown as comparator. All MOIs and mocks were performed side-by-side, and the mock is the same within panels for N, NF-κB and within the IRF3 panels. Horizontal lines indicate the mean. Kruskal–Wallis test with Dunn's multiple comparison, *$P < 0.05$; **$P < 0.01$; ***$P < 0.001$.

 © 2021 The Authors

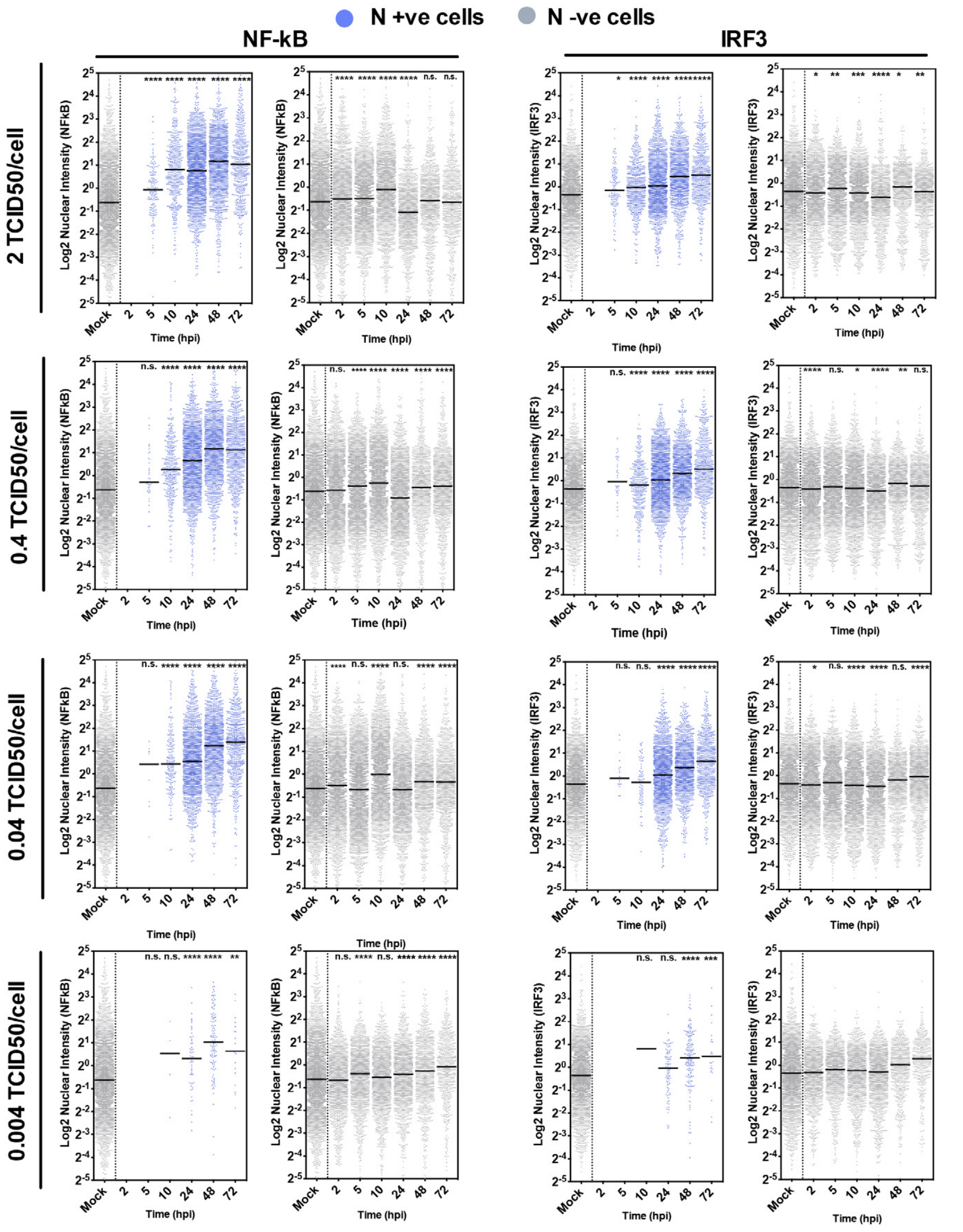
N +ve cells
N -ve cells
NF-kB
IRF3
2 TCID50/cell
0.4 TCID50/cell
0.04 TCID50/cell
0.004 TCID50/cell
Log2 Nuclear Intensity (NFkB)
Log2 Nuclear Intensity (IRF3)
Time (hpi)
Mock
2
5
10
24
48
72
n.s.

Figure EV3.

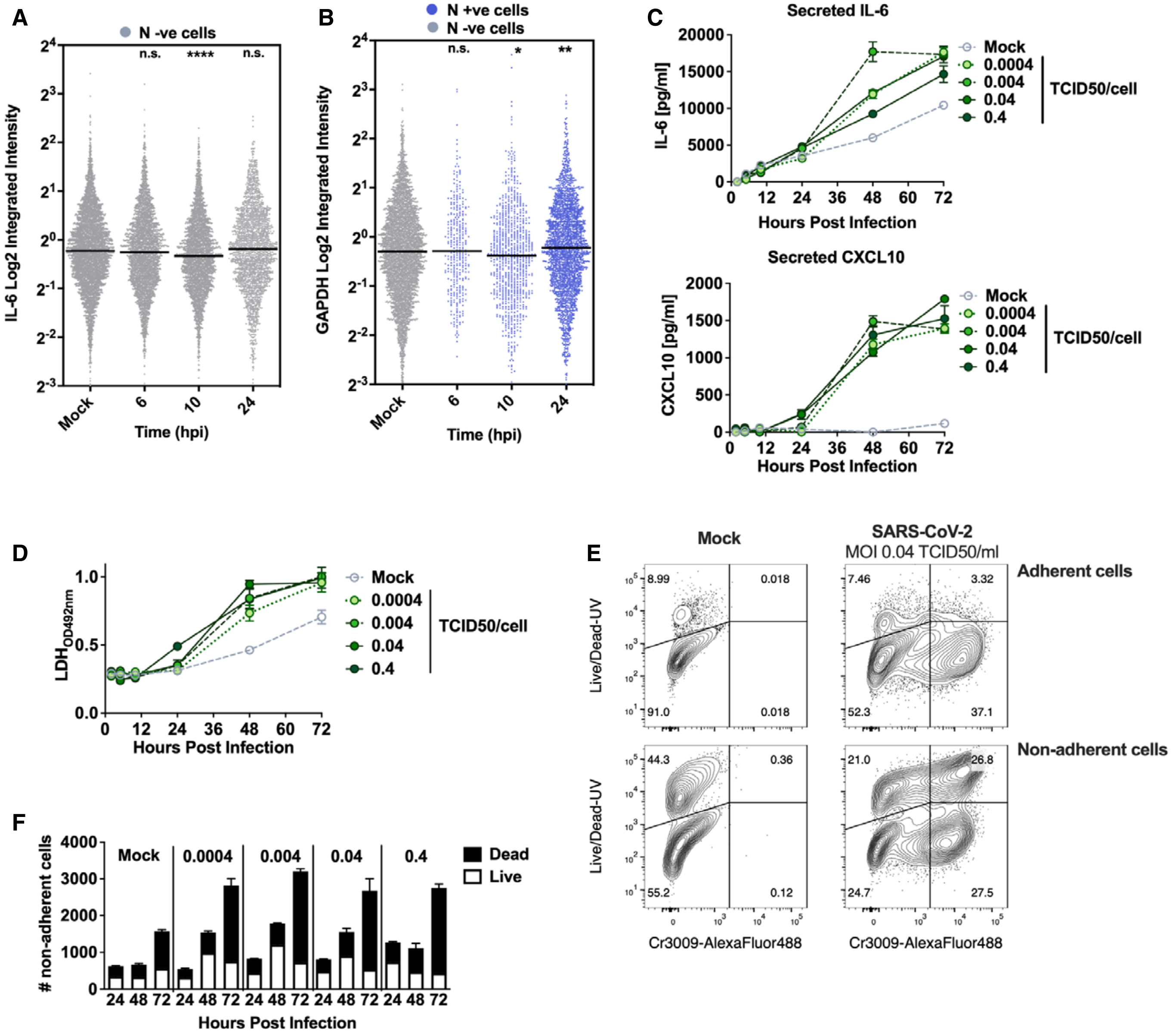

Figure EV4. SARS-CoV-2 activation of the innate response in Calu-3 cells coincides with inflammatory cell death.

A Representative single-cell RNA-FISH analysis time course quantifying the integrated intensity of *IL-6* in uninfected (Mock) or uninfected bystander cells (uninfected cells, grey) of Calu-3 cells infected at MOI 0.4 TCID50/cell.

B Representative single-cell RNA-FISH analysis time course quantifying the integrated intensity of *GAPDH* in uninfected (Mock), nucleocapsid protein-positive infected (blue) and uninfected bystander (grey) Calu-3 cells at MOI 0.4 TCID50/cell. (A, B) Horizontal lines indicate the median with Kruskal–Wallis test with Dunn's multiple comparison, $*P < 0.05$; $**P < 0.01$; $***P < 0.001$.

C Secretion of IL-6 and CXCL10 (ELISA) by infected Calu-3 cells (MOIs 0.0004, 0.004, 0.04 and 0.4 TCID50$_{VERO}$/cell), matching infections in Fig EV2A–C and F. Mean ± SEM, $n = 3$.

D Lactate dehydrogenase (LDH) release into culture supernatants by mock and SARS-CoV-2-infected Calu-3 cells (MOIs 0.0004, 0.004, 0.04 and 0.4 TCID50$_{VERO}$/cell, matching infections in Fig 2A–C and F) quantified by absorbance (492nm), means ± SEM, $n = 3$.

E Representative flow cytometry contour plots depicting intracellular nucleocapsid protein detection (Cr3009-AlexaFluor 488) and Live/Dead (Live/Dead-UV) staining. Shown are infected (MOI 0.04 TCID50 $_{VERO}$ /cell) and uninfected (Mock) Calu-3 cells at 48 h post-infection. Adherent and non-adherent cells were collected and acquired.

F Quantification of Live/Dead staining of non-adherent cells recovered from supernatants of Mock or SARS-CoV-2-infected Calu-3 cultures (MOIs 0.0004, 0.004, 0.04 and 0.4 TCID50$_{VERO}$/cell) at 24, 48 or 72 hpi. Mean ± SEM, $n = 3$.

 © 2021 The Authors

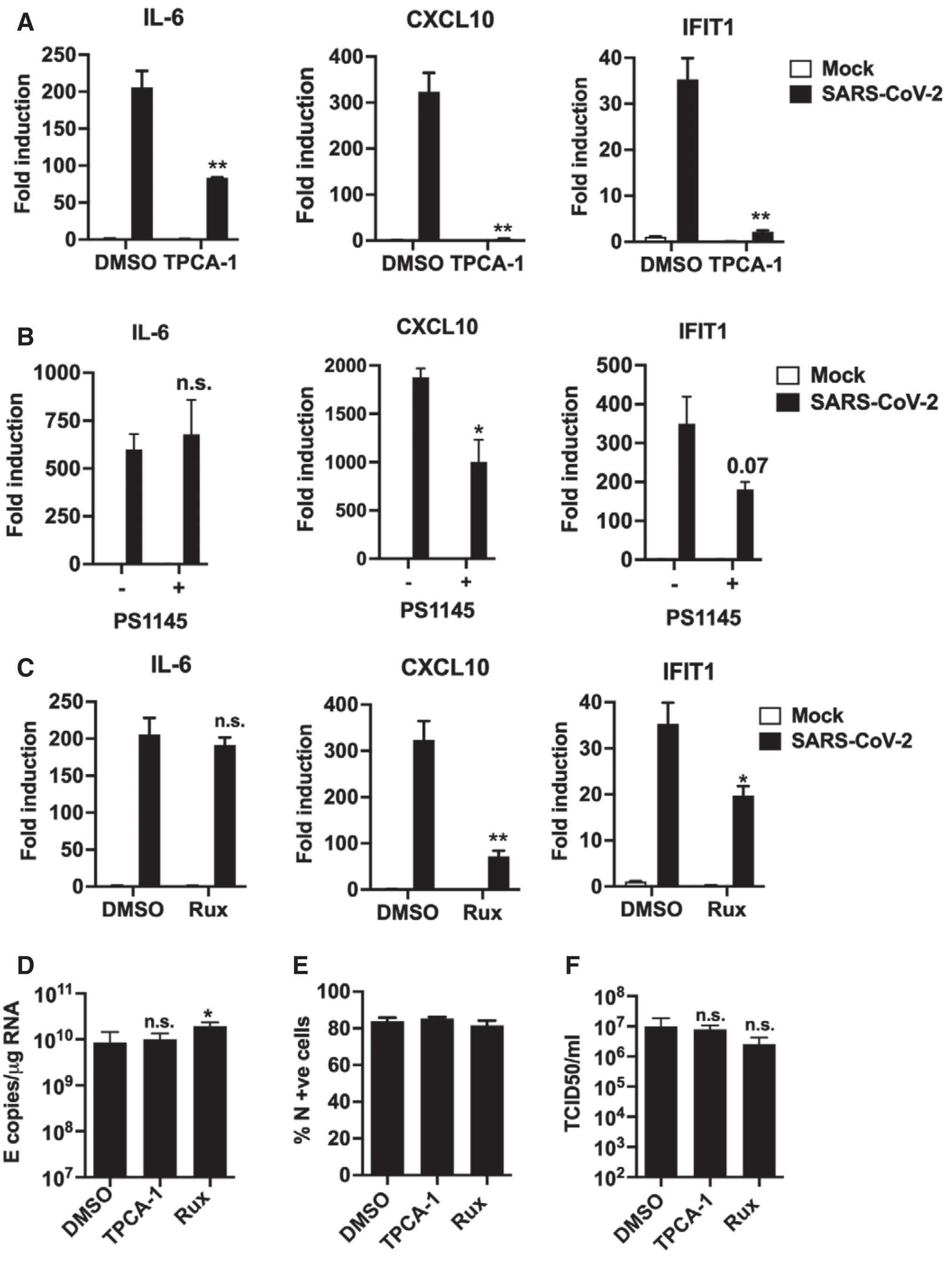

Figure EV5. Inhibition of IFN and NF-κB signalling reduces the inflammatory response during SARS-CoV-2 infection.

A–C Fold gene induction of *IL-6*, *CXCL10* and *IFIT1* 24 hpi of Calu-3 with SARS-CoV-2 (MOI 0.04 $TCID50_{VERO}$/cell) infected in the presence of: (A) 10 μM TPCA-1 (B) 10 μM PS1145 or (C) 10 μM Ruxolitinib (Rux) with DMSO as control in each case.

D–F Measurement of SARS-CoV-2 (MOI 0.04 $TCID50_{VERO}$/cell) replication in Calu-3 in the presence or absence of 10 μM TPCA-1, 10 μM Ruxolitinib (Rux) or DMSO vehicle as shown measuring (D) genomic and subgenomic E RNA, (E) N +ve cells by flow cytometry, (F) released virus in supernatant ($TCID50_{VERO}$/cell) at 24 hpi. Mock and SARS-CoV-2-infected/treated conditions were compared by two-tailed *t*-test. *$P < 0.05$; **$P < 0.01$; n.s., non-significant. Mean ± SEM, $n = 3$.
